# Patterns of local-regional recurrence after conformal and intensity-modulated radiotherapy for head and neck cancer

**DOI:** 10.1186/s13014-017-0829-5

**Published:** 2017-05-25

**Authors:** Safora Johansen, Mathilde H. Norman, Einar Dale, Cecilie D. Amdal, Torbjørn Furre, Eirik Malinen, Jan F. Evensen

**Affiliations:** 1 Department of oncology, Division of cancer Medicine, Surgery and Transplantation (KKT), Oslo University Hospital-Radium hospital, Montebello, 0310 Oslo, Norway; 2 Oslo and Akershus University College of Applied Sciences, Faculty of Health Sciences, Oslo, Norway; 3 Department of Physics, University of Oslo, Oslo, Norway; 4 Department of Medical Physics, Oslo University Hospital, Oslo, Norway

**Keywords:** Head and neck cancer, Radiotherapy, Radiotherapy technique, Regional lymph node, Recurrence, Relapse

## Abstract

**Aim:**

To evaluate the patterns of loco-regional recurrences in head and neck cancer patients

**Methods:**

Twenty-six out of 112 patients treated with primary or postoperative 3D CRT or IMRT for their primary and recurrent disease between 2007 and 2013 were included. The CT images of recurrent disease were rigidly registered with the primary CT images for each patient. To assess overlaps and overlap localization, the recurrence volume overlapping with the primary target volume was identified. For relapses occurring in the regional lymph nodes, the epicenter distance in recurrences and primary volumes and dose in recurrences were also identified. The recurrences were defined as in-field, marginal or out-of-field.

**Results:**

The majority of the failures occurred within 1 year after completed primary treatment. The dose differences in recurrence volume were not statistically significant when patients were treated with IMRT or 3D CRT. Recurrence in 15/26 of the included patients occurred in the regional lymph nodes located fully or partly inside the primary target volume or the elective lymph node region. The majority of recurrences were recognized as in-field, independent of the primary treatment.

**Conclusion:**

Recurrence in the majority of the patients occurred in the regional lymph nodes located in high dose area. The cause of recurrence may be due to inadequate total dose in the primary treatment and/or lack of optimal primary diagnosis leading to inadequate primary target delineation.

## Introduction

The incidence of oral cavity/pharynx and larynx cancer in Europe was estimated to 100,000 and 40,000, respectively, in 2012 [1]. In Norway, 800 patients were diagnosed with head and neck cancer (HNC) in 2014, representing 2.5% of the total incidence of malignant disease [2].

Management of HNC is multi-disciplinary; surgery, radiotherapy (RT) with or without concomitant chemotherapy. High dose RT (prescribing doses typically of 70 Gy) is necessary to achieve cure, but may result in side effects. The employment of three-dimensional conformal radiotherapy (3D CRT) and intensity-modulated radiotherapy (IMRT) have permitted treatment largely conforming to the disease extensions for the individual patient [3–6]. However, each year, 30–50% of patients with locally advanced HNC, experience loco-regional relapse [7]. Loco-regional recurrence is still one of the major causes of failure in HNC after radical treatment [7]. Therefore, it is important to evaluate the patterns of loco-regional recurrences in patients treated with 3D CRT and IMRT. Specifically, it is relevant to learn more about the cause of relapse, the dose delivered to the tissue in question and the proximity of the tumor recurrence to the original target structures. To address this issue, we have analyzed recurrence patterns in patients with recurrent HNC previously treated with 3D CRT and IMRT at our institution. A detailed mapping of the primary treatment, the patient dose distributions and subsequent recurrence patterns are provided.

## Methods

### Patients

We retrospectively reviewed the medical records of 112 patients with primary squamous cell carcinoma in the head and neck reirradiated between January 2007-December 2013. To be included in the current study the following criteria had to be fulfilled: i) re-irradiation for first relapse in the head and neck region, ii) radiotherapy for both primary and recurrent disease at Oslo University Hospital, iii) Computed Tomography (CT), and/or Positron Emission Tomography (PET)/magnetic resonance images (MRI) taken prior to RT, iv) available RT dose plans for the primary and recurrent disease which were technically possible to co-register, v) completed their planned curative primary radiation treatment.

Of the 112 patients, 26 patients fulfilled the inclusion criteria. The general characteristics of the patients and tumor site are detailed in Table 1. The patterns of recurrence were analyzed separately in 2 groups of patients based on the primary treatment they received; patients treated with primary RT (*n* = 10) and postoperative RT (*n* = 16). The time difference between primary RT and reirradiation varied between 4 and 63 months (Tables 2 and 3). At the time of recurrence, 16 of 26 patients had their gross tumor removed before reirradiation. In these patients, the gross tumor was delineated on the relapse RT CT images based on the diagnostic CT examination taken prior to relapse surgery.

**Table 1:** Patients characteristics

	Number of patients
Total	26
Median age	65.5(42–86)
Sex	
Male	20
Female	6
Postoperative radiotherapy	16
Primary radiotherapy	10
Chemotherapy	
Yes	2
No	24
Tumor site	
Oral cavity	13
Parotid gland	2
Epipharynx	4
(Case No. 2, 22 and 24)	
Larynx	4
Tonsilla	2
Nasal cavity	1
Radiotherapy technique	
IMRT	12
3D conformal	14

**Table 2:** Primary treatment history – Patients treated with primary RT

Case nr	Radiotherapy regimen	Radiotherapy technique	Treatment year	Time difference between first RT and recurrence RT (In months)	TNM
	Fraction D × No of fractions +/− concomitant boost (CB) or hyperfractionated RT (HF)/Total Dose	IMRT = I and Conformal = C			
1	2 Gy × 23 + CB 2 Gy × 12/70 Gy	I	2009	19	T4N1M0
2	HF 1.5 Gy × 20 + 1.5 Gy × 20/60 Gy	C	2009	14	T4N2bM0
3	2 Gy × 23 + CB 2 Gy × 12/70 Gy	I	2009	19	T2N2bM0
4	2 Gy × 23 + CB 2 Gy × 12/70 Gy	C	2008	41	T4N2aM0
5	1.5 × 15 + 1.75 × 4 + 1.5 × 20/59.5 Gy	I	2010	5	T4N0M1
6	2 Gy × 23 + CB 2 Gy × 12/70 Gy	C	2009	63	T2N0M0
7	2 Gy × 23 + CB 2 Gy × 12/70 Gy	C	2008	22	T3N0M0
8	2 Gy × 23 + CB 2 Gy × 11/68 Gy	I	2010	10	T4aNxM0
9	2 Gy × 23 + CB 2 Gy × 12/70 Gy	I	2009	18	T2N0M0
10	2 Gy × 23 + CB 2 Gy × 12/70 Gy	I	2009	10	T3N2bM0

**Table 3:** Primary treatment history – Patients treated with postoperative RT

Case nr	Radiotherapy regimen	Radiotherapy technique	Treatment year	Time difference between first RT and recurrence RT (In months)	TNM
	Fraction D × No of fractions +/− concomitant boost (CB) or hyperfractionated RT (HF)/Total Dose	IMRT = I and Conformal = C			
11	2 Gy × 23 + CB 2 Gy × 2/50 Gy	C	2010	33	T1N0M0
12	2 Gy × 23 + CB 2 Gy × 12/60 Gy	C	2007	43	T4N0M0
13	2 Gy × 25/50 Gy	C	2011	6	T1N0M0
14	2 Gy × 23 + CB 2 Gy × 10/66 Gy	I	2011	35	T2N0M0
15	2 Gy × 35/70 Gy	C	2010	10	T1N0M0
16	2 Gy × 23 + 2 Gy × 7 + 2 Gy × 3/66 Gy	I	2012	4	T2N2bM0
17	2 Gy × 23 + 2 Gy × 7/60 Gy	I	2011	6	T2N2bM0
18	2 Gy × 23 + 2 Gy × 7/60 Gy	C	2010	14	T1N2bM0
19	2 Gy × 23 + CB 2 Gy × 7/60 Gy	C	2009	10	T1N0M0
20	2 Gy × 23 + 2 Gy × 2/50 Gy	C	2012	6	T1N0M0
21	2 Gy × 23 + 2 Gy × 7/60 Gy	C	2010	7	T2N1M0
22	2 Gy × 33/66 Gy	I	2009	35	T4N0M0
23	2 Gy × 23 + 2 Gy × 2/50 Gy	I	2013	9	T2N0M0
24	2 Gy × 23 + 2 Gy × 2/50 Gy	I	2006	59	T3N2M0
25	2 Gy × 23 + 2 Gy × 7/60 Gy	C	2010	7	T2N2bM0
26	2 Gy × 23 + 2 Gy × 2/50 Gy	C	2010	5	T2N0M0

Only 2 of the 26 patients received concomitant chemotherapy (Table 1). For the primary treatment, 12 of 26 patients received IMRT while 14 patients were treated with 3D CRT for their HNC disease as reported in Tables 2 and 3.

### Tumor delineation

Tumor volumes were defined by an experienced radiation oncologist on simulation CT images acquired in conjunction with primary and recurrent RT, registered in some cases with MRI and/or Fluorodeoxyglucose (18 F-FDG) PET images. The gross tumor volume (GTV; GTVp for primary GTV and GTVr for recurrence GTV) was defined as the visible tumor based on all available diagnostic imaging as well as clinical examination. The high risk and standard risk areas with 10 mm margin to GTV was included in the clinical target volume (CTV, CTVp and CTVr). CTV also included non-dissected lymph nodes. The planning target volume (PTV, PTVp and PTVr) was constructed by expanding the corresponding CTV by 3 mm [8, 9].

### Image registration and overlap definition

**Fig. 1:**
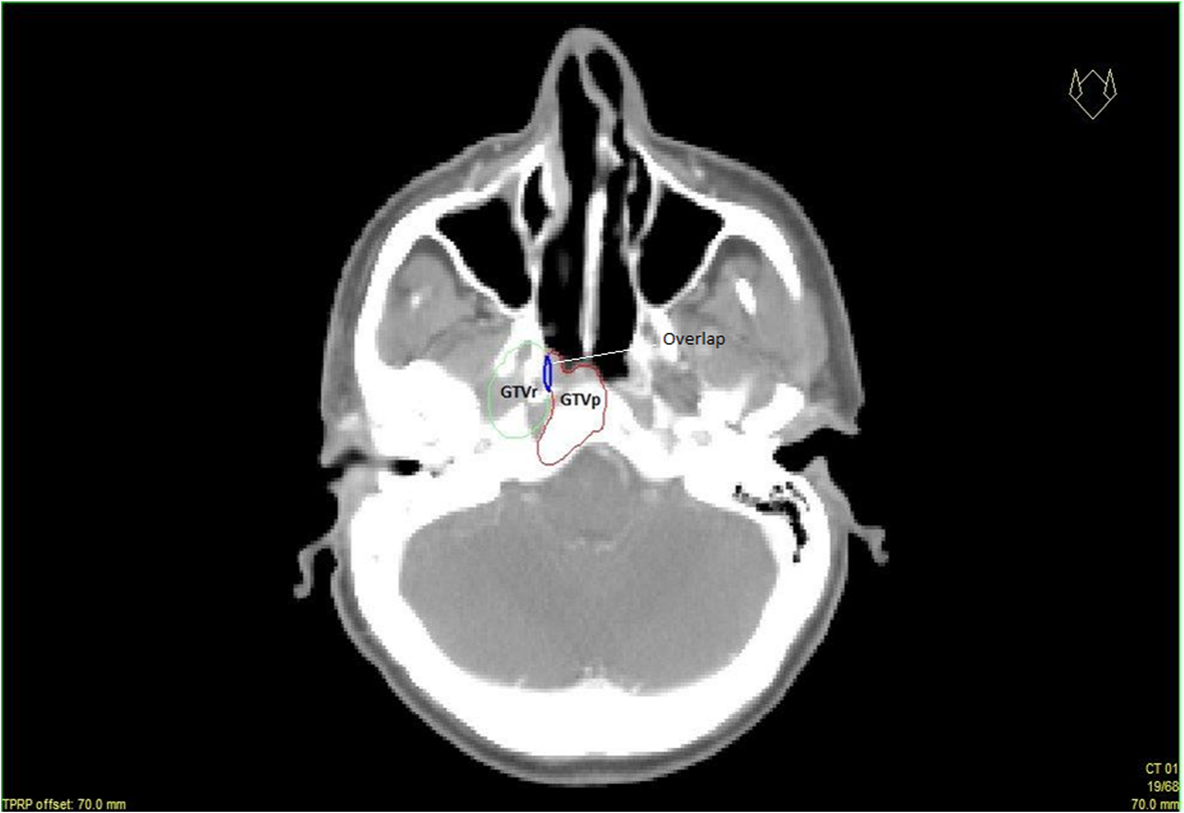
Fusion display of primary (gray) and recurrence (pink) CT images

Relapse localization on CT images was further investigated by using the software module Oncentra Masterplan (version 4.3). The recurrence treatment planning CT for each included patient was exported to the respective primary dose plan-CT series. The exported recurrence dose plan CT was further rigidly registered with the primary dose plan-CT for each patient using available image registration tools. To achieve an optimal image registration, the information such as skull base, frontal bone and other bony structures were used. After CT image registration, the GTVr from the recurrence dose plan-CT was copied and pasted into the primary dose plan CT dataset. The HNC oncologist (JFE) also approved the quality of image registration for each included patient (Fig. 1).

**Fig. 2:**
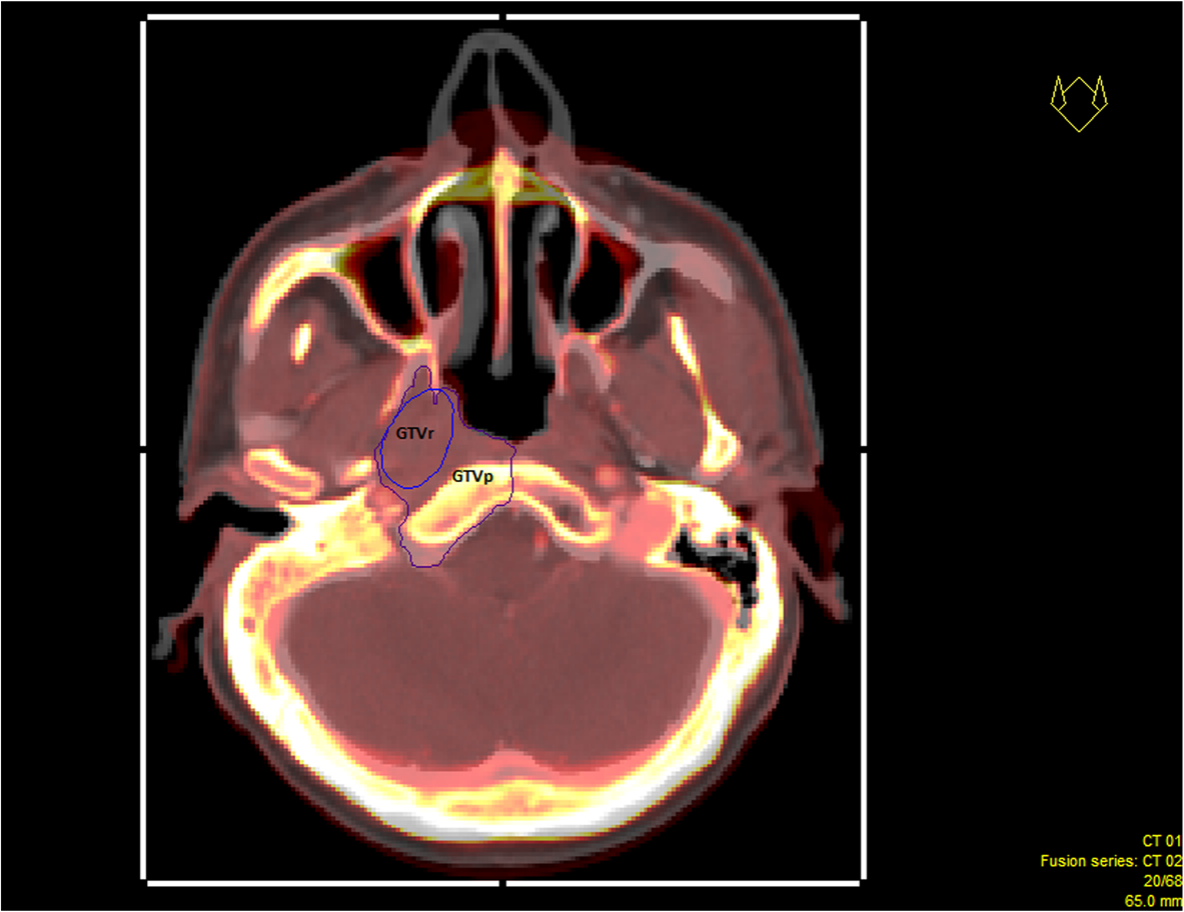
Definition of overlap volume

To assess overlaps, the GTVr overlapping with the GTVp in each primary CT slice was identified and delineated as illustrated in Fig. 2. For cases with relapses located fully or partly in the elective nodes, no overlap volume was assessed. Then the dose of both the primary RT plan and of the reirradiation plan in the overlap area was calculated.

To explain the cause of relapse, it was assessed whether the relapse occurred in the regional lymph nodes or not. The localization of the regional lymph node relapse was assessed by our oncologist.

The epicenter in GTVr and GTVp were defined, the dose and distance between the 2 epicenters were calculated. Epicenter was estimated from the center of mass. A mean epicenter distance value was calculated in cases with more than one GTVr.

The recurrence volumes were considered as in-field, marginal and out-of-field, if the dose in the epicenter of the recurrence volume was located in high, low or very low dose areas, respectively. Cases with their relapse located in 2 regional lymph nodes with different location, were labelled with 2 relapse site definitions but one mean epicenter dose.

### Statistics

To assess the dose differences when employing IMRT or 3D CRT, a two-tailed independent *T*-test was employed. A *p* value < 0.05 was considered as statistically significant.

### Ethics

All necessary approvals were obtained before the study was conducted. Written informed consent was obtained from all patients in accordance with the procedures of the Data Protection Office of whom also approved this study.

## Results

The range of the total prescribed RT dose for primary HNC treatment was 50–70 Gy, as shown in Tables 2 and 3. In total 13 (50%) of the failures occurred within 1 year after primary treatment, 6 (23%) within 2 years and 7 (27%) within 3 to 5 years. In patients treated with postoperative RT compared to those treated with primary RT, the fraction of the recurrences occurring within 1 year was approximately 3 times higher. The fraction of patients with relapse within 2 years was 4 times higher in the patient group treated with primary RT compared to postoperative RT patient group. The fraction of patients with relapse within 3–5 years after primary treatment was identical in both patient groups.

The population-averaged median dose to the recurrence volume for 26 patients receiving either IMRT or 3D CRT was 65.3, and 45.1 Gy, respectively (Data not shown). No statistically significant dose differences were observed between the employed RT techniques.

Recurrence occurred in the regional lymph nodes in 4 out of 10 patients treated with primary RT compared to 11 out of 16 patients treated with postoperative RT as shown in Tables 4 and 5.

**Table 4:** Site of relapse for patients with primary RT

Case No.	Site of relapse	Location of relapse in lymph node (yes = y or No = N)	Total relapse dose (Gy) as a result of first RT
1	Partly inside the primary tumor volume	N	71.0
2	Outside the primary tumor volume and elective lymph node volume	Y	27.0
3	Partly inside the primary tumor volume	Y	67.0
4	Partly inside the primary tumor volume and elective lymph node volume	Y	67.0
5	Partly inside the primary tumor volume	N	43.0
6	Fully inside the primary tumor volume	N	69.0
7	Partly inside the elective lymph node volume	Y	50.0
8	Fully inside the primary tumor volume	N	68.0
9	Partly inside the elective lymph node volume and touches the primary tumor volume	N	64.0
10	Partly inside the primary tumor volume and elective lymph node volume	N	70.0

**Table 5:** Site of relapse for patients treated with postoperative RT

Case No.	Site of relapse	Location of relapse in lymph node (yes = y or No = N or partly = P)	Total relapse dose (Gy) as a result of first RT
11	Relapse in a lymph node partly inside the elective lymph node volume and the other lymph node outside the elective lymph node volume and the primary tumor volume	Y	44.0 and 45.0
12	Partly inside the primary tumor volume	N	52.0
13	Fully outside the primary tumor volume and elective lymph node volume	Y	15.0
14	Partly inside the elective lymph node volume	Y	20.0
15	Fully inside the primary tumor volume	N	66.0
16	Partly inside the elective lymph node volume and primary tumor volume	N	60.0
17	Relapse in one lymph node volume partly inside the elective lymph node volume and another lymph node fully inside the elective lymph node volume	Y	9.0 And 47.0
18	Fully inside the elective lymph node volume	Y	59.0
19	Partly inside the elective lymph node volume	Y	60.0
20	Fully inside the elective lymph node volume	Y	38.0
21	Fully inside the elective lymph node volume	Y	45.0
22	Partly inside the primary tumor volume	N	66.0
23	Outside the elective lymph node volume and primary tumor volume	Y	2.0
24	Partly inside the primary tumor volume	N	50.0
25	Outside the elective lymph node volume and primary tumor volume	Y	9.0
26	Fully inside the elective lymph node volume and also touches the primary tumor volume	Y	41.0

Total relapse dose in patients who received primary RT varied from 27 to 71 Gy and in the patients treated with postoperative RT from 2 to 66 Gy (Tables 4 and 5). The dose in epicenters and distance between epicenters in GTVr and GTVp are shown in the Tables 4 and 5.

**Fig. 3:**
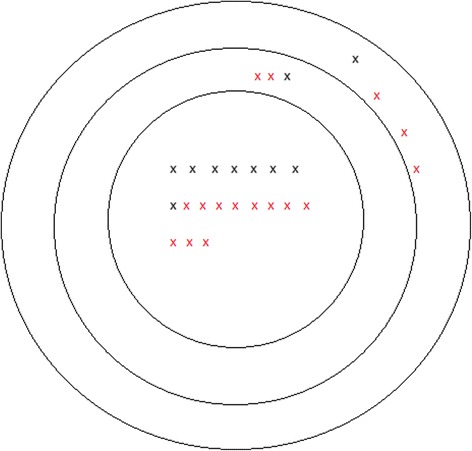
Schematic representation of 26 recurrences included in this study. Patients treated with primary RT and postoperative RT are shown with case number in black and red, respectively

There were 1 and 3 out-of-field recurrences in patients treated with primary RT and postoperative RT, respectively. One of 10 patients treated with primary RT and 2 of 16 patients treated with postoperative RT had marginal relapses (Tables 6 and 7 and Fig. 3). Eight and 11 in-field recurrences were identified in patients treated with primary RT and postoperative RT, respectively (Tables 6 and 7 and Fig. 3). The GTVr and GTVp epicenter distance in patients treated with primary RT and postoperative RT was 0.4–6.1 and 1.2–13.7 cm, respectively. The average epicenter distance for in-field, marginal and out-of-field recurrences were 3.8, 6.3 and 9.4 cm.

**Table 6:** Patient treated with primary RT; recurrence volume size, recurrence site, overlap volume size, epicenter dose and distance between epicenter in GTVr and GTVp

Case No.	Recurrence volume (ccm)	In-field	Marginal	Out-of- field	Overlap size (ccm)	Epicenter dose (Gy) in GTVr/GTVp and distance (cm)
1	6.5	X			4.0	71.4/70.2, 0.9
2	17.4			X	0.0	34.4/60.0, 5.2
3	73.7	X			17.1	69.2/71.2, 3.7
4	44.3	X			23.8	72.8/71.6, 1.7
5	76.0	X			18.3	56.2/61.5, 1.8
6	74.7	X			15.8	71.5/71.6, 0.4
7	41.1		X		0.0	54.7/72.4, 4.5
8	15.6	X			15.0	67.8/66.7, 3.0
9	46.4	X			7.3	70.5/71.2, 4.2
10	14.2	X			7.3	70.2/68.8, 2.8

**Table 7:** Patient treated with postoperative RT; recurrence volume size, recurrence site and overlap volume size, epicenter dose and distance between epicenter in GTVr and GTVp

Case No.	Recurrence volume (ccm)	In-field	Marginal	Out-of- field	Overlap size (ccm)	Epicenter dose (Gy) and distance (cm)
11	88.9	X			0.0/0.0	44/49.2, 6.6
12	24.2		X		1.5	48.8/61.5, 5.5
13	76.6			X	0.0	0.7/50.8, 12.0
14	18.0	X			0.0	66.4/65.2, 2.8
15	5.8	X			2.6	65.5/69.0, 2.9
16	86.6	X			37.7	64.3/61.7, 4.9
17	20.1		X		0.0/0.0	44.0/59.9, 8.8
18	5.2	X			0.0	63.0/58.6, 4.9
19	2.1	X			0.0	62.9/62.7, 5.2
20	18.2	X			0.0	45.3/50.4, 9.2
21	59.5	X			32.6	45.8/57.8, 9.3
22	13.5	X			9.5	65.2/69.7, 1.2
23	119.3			X	0.0	1.6/49.8, 13.7
24	27.2	X			4.3	52.7/52.7, 2.8
25	59.0			X	0.0	6.9/59.6, 6.7
26	27.1	X			1.3	46.4/49.7, 4.1

The size of recurrence volume varied between 6.5 to 76 ccm and 2.1 to 119.3 ccm in patients treated with primary RT and postoperative RT, respectively (Tables 6 and 7).

The mean overlap size for primary RT was 10.4 ccm (SD ± 8.6) compared to patient group receiving postoperative RT of 5.6 ccm (SD ± 11,8) (Tables 4 and 5) (*p* = 0.23).

## Discussion

This study analyzed the failure patterns of 26 HNC patients treated with RT for their relapse disease. Our analyses show that the majority of the failures occurred within 1 year after completed primary treatment. There was no statistical difference in the doses to the recurrence volume in patients treated with IMRT or 3D CRT technique. The majority of the recurrence in the patients treated with postoperative RT occurred in the regional lymph nodes. The recurrent lymph nodes were located fully or partly inside the elective lymph node region in the majority of these patients. The majority of recurrences were recognized as in-field, independent of the primary treatment.

Due et al. [10] have analyzed the recurrence pattern in 39 HN squamous cell carcinoma patients with loco-regional failure treated with chemoradiation. Due and colleagues [10] reported that 96% of recurrences were located in the high dose region. Another study carried out by Soto et al. [11] showed that 100% (9/9) of the included HNC patients with loco-regional failure were located inside the primary GTV. The shorter average epicenter distance of 3.8 cm between GTVr and GTVp, the high epicenter dose in GTVr and the reported site of recurrences in this study show that the majority of recurrences (73%) are also located in high dose area in accordance with the studies of Due et al. and Sot et al. [10, 11].

In the study of Due et al. [10] all the included patients were treated with primary RT, while in the current study only 10 of the included patients received primary RT and 16 patients were treated with postoperative RT. Our results showed no correlation between the recurrence patterns and the kind of primary treatment the patients had received. The cause of relapse in the study of Due et al. [10] seems to be insufficient total dose to the primary GTV. However, in the present study the relapse could be due to: i) inadequate total dose to the primary target volume and/or ii) imprecise diagnostic imaging of the primary tumor volume leading to inadequate primary target delineation. In the majority of the cases (15/26) in our study, regional recurrences occurred in lymph nodes partly or fully located in the area of the primary GTV or elective lymph nodes. Therefore, it may be questioned whether the total dose in this area was adequate or not. In the marginal and out-of-field recurrences the cause of relapse seems to be due to the insufficient primary target delineation. Increased use of FDG-PET in defining the GTV as discussed in the study of Soto et al. [11] could probably improve the accuracy of the tumor definition.

IMRT and 3D CRT techniques are usually employed in the standard management of HNC patients. In only one of the marginal recurrences in this study IMRT technique was used in the delivery of their primary RT. Increased risk of marginal misses is often mentioned as one of the disadvantages associated with IMRT [12]. Precise target definition is therefore crucial to avoid marginal miss.

One limitation of the current study is the heterogeneity of the patient cohort and their stage of disease. However, it is important to stress that the goal of this study was to assess the relapse localization and the cause of it. Another limitation is that the CT slice thickness in primary and recurrence CT scans were not always identical. This may have resulted in less precise fusion of the primary and relapse CT scans. In general, some uncertainty can be associated with the rigid registration process because of anatomical changes between the primary and recurrence RT. However, our experienced oncologist has studied the detailed disease information in each medical record and diagnostic CT images and compared them with the recurrence localization assessed after registration. Another limitation when using rigid registration is inaccurate estimate of the dose distribution in the CT series of the recurrence volume.

## Conclusion

The cause of recurrence in the majority of the patients seems to be inadequate dose to the primary treatment volume and in some few cases due to imprecise primary diagnostic imaging leading to inadequate primary target delineation. The majority of recurrences were recognized as in-field, independent of the primary treatment. Further investigation is necessary to evaluate the total RT dose needed to treat the primary HNC. Optimal diagnostic methods should be employed to avoid poor primary target delineation.

## Abbreviations

18 F-FDG: Fluorodeoxyglucose
3D CRT: Three-dimensional conformal radiotherapy
CT: Computer tomography
CTV: Clinical target volume
GTV: Gross tumor volume
HNC: Head and neck cancer
IMRT: Intensity-modulated radiotherapy
MRI: Magnetic resonance images
PET: Positron emission tomography
RT: Radiotherapy
